# The effect of intraarticular levobupivacaine and bupivacaine injection on the postoperative pain management in total knee artroplastic surgery

**DOI:** 10.12669/pjms.306.5877

**Published:** 2014

**Authors:** Nurcan Yavuz, Vildan Taspinar, Derya Karasu, Aysu Tezcan, Bayazit Dikmen, Nermin Gogus

**Affiliations:** 1 Nurcan Yavuz, MD, Assistant Professor, Department of Anesthesiology and Reanimation, Ankara Numune Training and Research Hospital, Ankara, Turkey.; 2 Vildan Taspinar, MD, Chairman, Department of Anesthesiology and Reanimation, Ankara Numune Training and Research Hospital, Ankara, Turkey.; 3 Derya Karasu, MD, Assistant Professor of Anesthesiology and Reanimation, Sevket Yilmaz Training and Research Hospital, Turkey.; 4 Aysu Tezcan, MD, Assistant Professor, Department of Anesthesiology and Reanimation, Ankara Numune Training and Research Hospital, Ankara, Turkey.; 5 Bayazit Dikmen, MD, Assistant Professor, Department of Anesthesiology and Reanimation, Gazi University Faculty of Medicine, Ankara, Turkey.; 6 Nermin Gogus, MD, Chairman, Department of Anesthesiology and Reanimation, Hitit University Faculty of Medicine, Corum, Turkey.

**Keywords:** Total knee arthroplasty, Postoperative analgesia, Patient controlled epidural analgesia, Intraarticular

## Abstract

***Objective:*** Total knee arthroplasty (TKA) is associated with considerable postoperative pain. We compared the effects of intraoperative intraarticular levobupivacaine and bupivacaine on postoperative analgesia and analgesic consumption after total knee arthroplasty.

***Methods:*** Sixty ASA (American Society of Anesthesiologists) physical status II-III, 18-75 years old patients scheduled for unilateral TKA were included in this study. For the operative procedure combined spinal epidural anesthesia was given by injecting 15mg levobupivacaine in subarachnoid space at L_3-4_/L_4-__5 _in sitting position for all patients. In Group L 20ml levobupivacaine(0.5%), in Group B 20ml bupivacaine (0.5%) was injected intraarticularly 10 minutes before opening of the tourniquet at the end of the surgery. For all patients postoperative analgesia was provided with PCEA (levobupivacaine+fentanyl) and oral 1gr paracetamol four times a day. Patients’ intraoperative-postoperative hemodynamical data, postoperative sensorial-motor block characteristics, side effects, PCEA demand ratios and bolus volumes, total analgesic consumption, VAS values, first mobilization time, hospitalization time were recorded. Statistical analysis was performed with SPSS version 13.00 software.

***Results:*** There was no intergroup difference in demographic data, hemodynamical data, PCEA demand ratios, total analgesic consumption, first mobilization time, hospitalization time and VAS values at 0,2,72 hour. Postoperative lower VAS values were determined at 4,8,12,24 hours in Group B and at 48^th^ hour in Group L(p<0.05).

***Conclusions:*** Intraarticular local anesthetic administration in addition to PCEA for post operative pain relief provides good analgesia after TKA surgery.

## INTRODUCTION

Total knee arthroplasty may be carried out under regional or general anesthesia. While general anesthesia provides better muscle relaxation, there is less bleeding, and less impact on mental status in regional anesthesia (spinal, epidural, combined spinal-epidural anesthesia). Regional methods reduce the risk for deep vein thrombosis and provide postoperative analgesia. Managing intraoperative and postoperative pain related to total knee arthroplasty surgeries is of particular concern to orthopedist as well as anesthesiologists.^1^^,^^2^ Patients suffer from severe pain, edema, and spasms in the early postoperative period after total knee arthroplasty operations. Postoperative analgesia has significant role in increasing joint mobility, improving muscle strength and providing mobilization after surgery. Systemic opioids, epidural analgesia with opioids, local anesthetics, and peripheral nerve blocks may be used for management of postoperative pain. Levobupivacaine is a *S(—*)-enantiomer of the racemic formulation of bupivacaine. Levobupivacaine is preferred as an alternative long acting local anesthetic to bupivacaine. An optimal local anesthetic for neural blockade must have a short onset time, a long duration of blockade, and minimal side effects.^3^ When compared to bupivacaine, levobupivacaine appears to have a larger margin of safety in terms of cardiovascular and central adverse effects when used in large doses.^4^ Although intravenous methods can provide better analgesia at rest, they fail to ensure effective relief for motion-related pain and to prevent reflex spasms in the quadriceps muscles.^5^

The aim of the present study was to compare bupivacaine and levobupivacaine administered as intraoperative intraarticular injections in knee arthroplasty operations in terms of analgesic consumption volumes and effects on postoperative pain. 

## METHODS

After institutional ethical committee approval, each patient’s written informed consent was obtained. Sixty patients scheduled for total knee arthroplasty (TKA) were screened for eligibility. Patients were randomly allocated to two groups according to a computer-generated list of random numbers that were placed in opaque sealed envelopes. 

The inclusion criteria were: age 18 to 75 years old, American Society of Anesthesiologists (ASA) physical status classification between (II-III), and normal preoperative mobility. Exclusion criteria included cardiovascular, hepatic, renal, neurologic, allergic, or endocrine diseases; pregnancy or breastfeeding; alcohol and substance addiction; allergy to local anesthetics; coagulopathy; hearing loss; treatment for chronic pain; and nervous system diseases. 

Patients were informed about the procedure and their questions were answered duiring the preoperative visit on the day before surgery. All patients were informed about the use of the patient-controlled epidural analgesia (PCEA) equipment and the Visual Analog Scale (VAS: 0=no pain, 10= unbearable pain). Forty five minutes before the operation, patients were preloaded with Ringer's lactate solution 10ml/kg. 

Routine monitors applied in the operating room included electrocardiograph, noninvasive blood pressure, and pulse oximeter (KMA275; Petas, Ankara-Turkey). Values obtained before the placement of the block were recorded. 0.03 mg/kg iv midazolam was administered for sedation before the procedure.

The patient was placed in sitting position, the insertion area was prepared using antiseptic solutions, and 2-3 ml lidocaine 2% was injected into the skin and subcutaneous tissue for local anesthesia. The epidural space was detected by the loss of air resistance after forwarding a combined spinal needle set (Portex 18/27 gauge, UK) through the L3-4 or L4-5 disc spaces. Afterwards, the subarachnoid space was reached. After observing the free flow of cerebrospinal fluid, 15 mg levobupivacaine 0.5% (Chirocaine ® 0.5% levobupivacaine hydrochloride 5 mg/ml, 10 ml Abbott, Istanbul, Turkey) was injected into subarachnoid space for 30 seconds. After injection, the spinal needle was removed and an epidural catheter was inserted through the epidural needle and advanced downward 2–3 cm into the epidural space. The operation was started when the spinal block reached the suitable level. During the operation, the patients were given O2 through the Vent mask at a rate of 3 lt /min.

Systolic, diastolic, and mean blood pressure, heart rate and oxygen saturation (SpO2) were being recorded from the moment that patients were taken to the operating room till the end of the operation (at 5 minute intervals during the first 30 minutes, then at 15 minute intervals from the 30th to 90th minutes, and at 30 minute intervals during the rest of the operation).

Presence of nausea-vomiting, pruritus, bradycardia, and hypotension was followed up in perioperative and postoperative periods, and patients were respectively administered metoclopramide (0.25 mg / kg iv), naloxone (0.2 mg iv), atropine (0.015 mg / kg iv) and ephedrine for treatment. Presence of urinary retention was examined through the postoperative follow-up.

Prior to the opening of the tourniquet, the surgeon administered 20 ml local anesthetics, bupivacaine 0.5% (Marcaine ® 0.5% bupivacaine hydrochloride 5 mg / mL, 20 mL, AstraZeneca, Istanbul, Turkey) for Group B and levobupivacaine 0.5% (Chirocaine ® 0.5% levobupivacaine hydrochloride 5 mg/ml, 10 ml Abbott, Istanbul, Turkey) for Group L, on the area where knee arthroplasty would be applied. In the postoperative period, we used standard patient-controlled epidural analgesia (PCEA) protocol using a mixture of levobupivacaine (0.125%) and fentanyl (2 mcg / mL) through the epidural catheter with a bolus dose of 5 ml and lockout period of 30 minutes (without loading dose and no 4 hourly maximum dose limit). All patients received epidural analgesia via the same type of PCA (Patient Controlled Analgesia) device (Abbott; Pain Management Provider, Chicago, Il, USA). Patients were given 1 g oral paracetamol every 6 hour in the postoperative period.

Patient data regarding intraoperative and postoperative hemodynamic variables, sensory and motor characteristics of the block at the 2nd, 4th, 8th, 12th, 24th, 48th, and 72th postoperative hours, side effects if any, the number of requested boluses and bolus doses for PCEA, the total amount of analgesics, VAS scores, the first mobilization and discharge time was recorded.

Presence of pain, if any during physiotherapy, was also recorded in follow-up. Isometric quadriceps exercises were started on the first postoperative day. After the removal of the drainage on the surgical site at the postoperative 48th hour, patients started isotonic quadriceps exercises (hip flexion during knee flexion) as well as the exercises they could do at the edge of the bed.

Twelve hours before surgery, all the patients received low-molecular-weight heparin as prophylaxis against thromboembolism. Prophylaxis was maintained in the postoperative period, as well. In all patients, the epidural catheters were withdrawn at the end of the postoperative 72th hour and 12 hours after the last dose of LMWH (Low Molecular Weight Heparin). Antibiotic prophylaxis started in the preoperative period was continued through the postoperative period.

The anesthetists analyzing characteristics of sensory and motor block, and side effects, conducting postoperative follow-up, and applying subarachnoid injection were different and not informed of the study.

***Statistical Method:*** Statistical analysis were carried out with the SPSS 13.00 Windows software program. The mean, standard deviation, median (minimum-maximum) values and percentage were used to express the obtained data. The analysis of variance was utilized to determine whether the average of the variables analyzed in the study differed between the groups or not. The dependent t test was used to detect whether there were any statistically significant difference between the dependent variables. Additionally, the Chi-square test was applied to see whether the distribution of categorical variable showed any difference between the two groups or not. Prestudy power analysis using our patient population mean and standard deviation suggested that 25 patients in each group (power of 90%) would be sufficient. Considering the number of patients lost to follow up and withdrawal cases, 75 patients scheduled for TKA were enrolled. While p<0.05 was considered significant, p<0.01 was used to indicate high significance. 

## RESULTS

The study was initiated with 75 patients; however, 8 patients rejected the procedure, epidural catheters could not be placed in 2 patients, and epidural catheters of other 5 patients displaced in the postoperative period. Consequently, a total of 60 patients (30 patients from Group B and 30 patients from Group L) were included in the study. No statistically significant difference was observed between the groups regarding age, weight, and height of patients, gender distribution, ASA status, and the duration of the operations (p>0.05) (Table-I).

There was no statistically significant difference in either group in terms of heart beat, systolic, diastolic, and mean arterial pressure, oxygen saturation values during the operation (p>0.05).

Additionally, we did not observe any significant difference between the groups regarding nausea-vomiting, pruritus, respiratory depression, bradycardia, and urinary retention during the postoperative period (p>0.05). In the postoperative period, one patient from Group L had hypotension at the 1st hour while one patient from Group B experienced hypotension at the 2nd hour. Also, two patients (one from each group) were reported to develop hypotension at the 4th hour.

Motor block carried on in all patients of both groups at the postoperative 0th hour. At the postoperative 2nd hour, 12 patients from Group B and 7 patients from Group L; at the postoperative 4th hour 2 patients (one from each group) suffered from motor block. However, none of the patients had motor block at the postoperative 8th hour.

**Fig.1:**
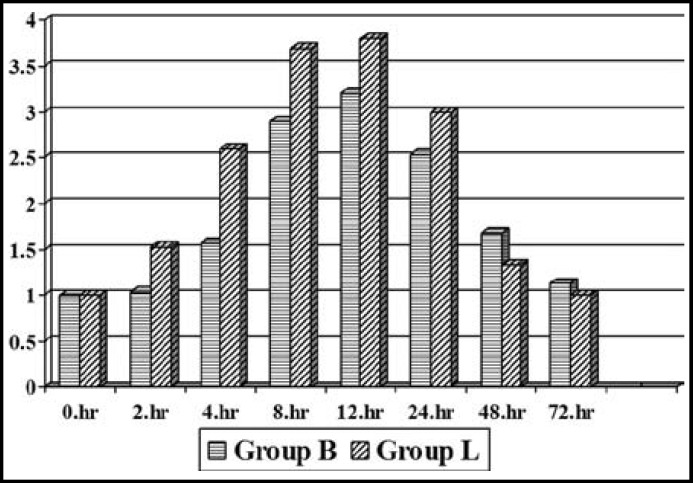
Visual Analog Scale (VAS) scores.

In the study, the VAS scores at the 0th, 2nd, and 72th hours did not show any significant difference between the groups. Whereas, the VAS scores obtained at the 4th, 8th, 12th, and 24th hours were lower in Group B and the VAS scores obtained at the 48th hour were lower in Group L (p<0.05) (Fig.1).The number of requested and applied PCEA boluses, the total amount of analgesics (Table-II), and time of the first mobilization and discharge were similar for both groups. 

In neither of the groups, there was a statistically significant difference between the number of requested boluses, applied bolus doses, and total dose used for patient-controlled epidural analgesia at the 1st, 2nd, and 3th postoperative hours.

There was no statistically significant difference between the pain scores recorded during postoperative physiotherapy (24th, 48th, and 72th hours) (p>0.05) (Table-III). It was observed that when the first mobilization time was compared between the patients, 6 patients from each group became mobilized at the 48th hour while the other 24 patients from each group regained their mobility at the 72th hour. Furthermore, when compared, the discharge days were not significantly different (p>0.05) between two groups.

## DISCUSSION

In the present study, we administered intraarticular injection of local anesthetics and paracetamol (1 g orally every 6 h), and applied PCEA to prevent postoperative pain after total knee arthroplasty. The VAS scores at the 4th, 8th, 12th, and 24th hours were lower in Group B and the VAS scores obtained at the 48th hour were lower in Group L. The number of requested PCEA boluses and applied bolus doses, total amount of analgesics, and pain scores during physiotherapy, mobilization and discharge times were similar for both groups. 

The stress response to surgical trauma and postoperative pain delay the recovery of patient, and increase the mortality and morbidity. Pain is one of the significant problems experienced after major orthopedic surgeries, such as total knee arthroplasty, and thus, different methods and medications are used to manage postoperative pain.^6^^,^^7^ However, optimal control of postoperative pain cannot be achieved in 50% of patients.^8^ Intraarticular pain treatment after TKA reduced pain and increase mobilization, and thereby increased range of motion and reduced hospital stay as these medications provide inhibition of inflammatory response and suppress the neuroendocrine stress response.^9^

The factors affecting the pain after total knee arthroplasty and the quality of intraarticular analgesia are; preoperative pain score, type of anesthesia, type of surgical procedure, duration of the operation, quality of the operation, intraarticularly injected agent and its volume.^10^ As such details were not recorded in many studies and researchers used different analgesics as premedication and in the perioperative period, it is hard to compare our study with earlier studies. It is important to standardize the method applied while assessing the efficacy of analgesics.^11^ While some researchers analyzed the pain experienced only in rest position, some preferred to examine the pain scores both at rest and motion.^12^ In this study, we analyzed pain at rest and during physiotherapy within the 72-hour postoperative follow-up. 

**Table-I:** Patient characteristics and intra-operative data in the two groups †

**Variations**	**Group B (n=30)**	**Group L (n=30)**	**P value**
Age (years)	65.23±6.66	65.9±7.45	0.716
Weight (kg)	81.07±10.25	81.9±8.43	0.732
Height (cm)	160.47±5.74	159.9±4.38	0.669
Gender (M/F)	26/4	24/6	0.488
ASA II/III	16/14	19/11	0.432
Duration of surgery (min)	92.17±18.50	97±19.36	0.327

American Society of Anesthesiologists: ASA

† Values are the number of patients (n) or means ±SD

* P<0.05 between groupsNo significant differences were found between the two groups

**Table-II:** Postoperative Assessment with PCA†

**Variations**		**1**^st^** Day**	**2** ^nd^ ** Day**	**3** ^rd^ ** Day **	**P value**
Number of boluses required	Group B	31.47±24.39	24.07±20.25	19.20±14.59	0.084
	Group L	38.87±28.27	21.13±16.82	17.77±12.38	
Number of boluses delivered	Group B	15.87±6.66	9.97±4.79	8.93±3.95	0.348
	Group L	16.37±8.39	9.70±5.42	8.50±4.20	
Total Consumption(ml)	Group B	79.17±33.45	49.67±23.81	44.67±19.78	0.371
	Group L	81.67±41.65	48.5±27.13	42.5±20.99	

PCA: Patient Controlled Analgesia.

† Values are the means ±SD.

* P<0.05 between groups.No significant differences were found between the two groups.

**Table-III:** The patients’ satisfaction about the pain control in 72 hours postoperatively physiotherapy†

**Variations**		**Excellent**	** Good**	** Poor**
Group B (n=30)	Postoperative 24^th^ hours	23	6	1
	Postoperative 48^th^ hours	3	25	2
	Postoperative 72^th^ hours	10	20	-
Group L (n=30)	Postoperative 24^th^ hours	21	8	1
	Postoperative 48^th^ hours	-	25	5
	Postoperative 72^th^ hours	2	25	3

† Values are the number of patients (n).

Several factors must be taken into consideration before intraarticular administration of analgesics. First, the intraarticular application should yield better results compared to systemic application for the same amount of analgesic. Second, drug diffusion should remain limited within the joint with minimal plasma absorption and have limited systemic effect. Third, the influence of analgesics should be supported with local mechanism. Intraarticular injections of local anesthetics provide quicker anesthesia and analgesia but anesthesia and analgesia last relatively for shorter period. However, high systemic absorption and toxic concentration levels impose serious restriction on the dose of medication to be used for analgesia.^12^ Another important point to be considered is that applications with continuous infusion pose an increasing risk for infection.^9^ Despite all these, as postoperative pain after arthroplasty originate in joints, it is logical to administer peripherally acting agents to the damaged area. Moreover, as applied doses of the agent have minimum or very few side effects, the usage is increasing day by day.^13^

 There is no consensus among researchers regarding the dose of intraarticular analgesic agents and whether they should be used alone or in combination. Bupivacaine is the most commonly used local anesthetic in postoperative pain treatment, and levobupivacaine has recently started being used as an alternative to bupivacaine. Clinical studies have indicated that anesthetic and / or analgesic effects of levobupivacaine are substantially similar to the effects of the same dose of bupivacaine. Animal studies have showed that levobupivacaine has lower cardiac and systemic toxicity compared to bupivacaine.^14^ However, there is only a limited number of publications regarding intraarticular administration of levobupivacaine for postoperative analgesia. It has been indicated that local anesthetics commonly used for treatment of postoperative pain experienced after arthroscopic surgery may damage cartilage tissue.^15^ Nonetheless, it seems a common belief that such a damage may occur when high doses of local anesthetics are used combined with corticosteroids for a longer timer.^16^^.^^17^ In this study, we injected a single dose of local anesthetics intraarticularly without corticosteroids, and thus, tried to reduce the risk for cartilage damage. 

Stein et al. have reported that there is direct correlation between the length of operation and postoperative pain level in the patients undergoing arthroscopic knee surgery.^18^ However, although there was no difference between the groups regarding duration of operations, the VAS scores were different in the present study. In this study, while there was no significant difference between the groups regarding the VAS scores at the 0th, 2nd, and 72th hours, the difference was significant at the 4th, 8th, 12th, 24th, and 48th hours. The VAS scores at the 4th, 8th, 12th, and 24th hours were found to be lower in the bupivacaine group (Group B) in comparison to the levobupivacaine group (Group L). But the VAS scores recorded at the postoperative 48th hour were lower in Group L as compared to Group B. There was no difference between the groups at the 0th and 2nd hours and the VAS scores were very low during that period. It may be due to the lingering effects of spinal anesthesia in both groups. The highest VAS scores were recorded at the 8th and 12th hours. Using levobupivacaine in the PCEA mixture may have had additional effect on the gradual decrease in VAS scores especially seen in the Group L after the 12th hour. Additionally, despite passive and active exercises started as of the 24th hour, the VAS scores kept decreasing. That may be also result of PCEA application.

In the study comparing postoperative analgesia methods (PCEA) in patients having major orthopedic surgery, Kopacz et al. administered epidural bolus infusion of fentanyl (4 mcg / mL) to the first group, of levobupivacaine (0.125%) to the second group, and of a combination of levobupivacaine (0.125%) + fentanyl (4 mcg / mL ) to the third group. In their study the analgesic requirement and VAS scores were lower in the group receiving the two-drug combination.^19^ In the present study, we applied PCEA in both groups with a bolus of levobupivacaine (0.125% ) + fentanyl ( 2 mcg / mL) combination depending on the patient's request in the postoperative period. Despite administration of lower and bolus dose for PCEA, the VAS pain scores were 4 or below 4. This may also be attributed to the intraarticular infusion of local anesthetics. 

As most of the studies regarding the intraarticular infusion of levobupivacaine and bupivacaine included patients undergoing outpatient total knee arthroplasty, postoperative follow-up lasted just up to 24 or 48 hours.^20^ In our study, however, patients were followed up during postoperative 3 days, and their VAS scores were recorded during that period. It was, therefore, difficult to compare the data recorded at the postoperative 48th and 72th hours with other related studies.

Bozkurt et al. have reported that epidural PCA is superior to intravenous PCA regarding management of pain both in the postoperative period and during knee rehabilitation after total knee arthroplasty.^2^ Ong et al. divided patients into 3 different groups for pain management after total knee arthroplasty. First group was applied PCA, while the second group received PCA together with continuous intraarticular and subcutaneous local anesthetic infiltration, and the third group was injected intraarticular local anesthetics as well as PCA with continuous intraarticular and subcutaneous local anesthetic infiltration. Consequently, they observed higher VAS scores in the first group.^10^

Bengisun et al. applied postoperative multimodal analgesia in patients having total knee arthroplasty. In their study, they recorded lower VAS scores and shorter hospitalization in patients receiving intraarticular infusion of levobupivacaine or bupivacaine with epinephrine for PCA compared to the control group.^4^

A limitation of this study is that the plasma concentrations of analgesics were not measured, and researchers did not have information on whether the drugs could develop any condrotoxic effect or not. 

Treatment of pain after total knee arthroplasty, which is related to severe postoperative pain, is very important for postoperative rehabilitation. We believe that applying intraarticular local anesthetics used for postoperative pain relief together with the PCEA method can provide effective analgesia. Our results point to the utility of a structured surgery-specific pain management protocol, which also benefits from a multimodal approach. Multimodal analgesia in the setting of TKA may incorporate nerve blockade, intravenous and peroral medications as well as a coordinated rehabilitation program.

## Authors’ Contribution:

**NY, VT, and BD** conceived, designed the study and prepared the manuscript. **NY and DK** supplied materials besides other contributions. **NY and VT** did statistical analysis and review. **NY, VT and AT** was involved in literature search. **NY and VT **did data collection and manuscript writing. **NY, VT, BD and NG** did review and final approval of manuscript.

## Notes

**Financial support**: Support was provided from institutional and departmental sources.

## References

[1] Sitsen E, Van Poorten F, Van Alphen W, Rose L, Dahan A, Stienstra R (2007). Postoperative epidural analgesia after total knee arthroplasty with sufentanil 1 microg/ml combined with ropivacaine 0.2%, ropivacaine 0.125%, or levobupivacaine 0.125%: a randomized, double-blind comparison. Anesth Pain Med.

[2] Bozkurt M, Yilmazlar A, Bilgen OF (2009). Comparing the effects of analgesia techniques with controlled intravenous and epidural on postoperative pain and knee rehabilitation after total knee arthroplasty. Joint Diseases and Related Surgery.

[3] Baskan S, Taspinar V, Ozdogan L, Gulsoy KY, Erk G, Dikmen B (2010). Comparison of 0.25% levobupivacaine and 0.25% bupivacaine for posterior approach interscalene brachial plexus block. J Anesth.

[4] Bengisun ZK, Salviz EA, Darcin K, Suer H, Ates Y (2010). Intraarticular levobupivacaine or bupivacaine administration decreases pain scores and provides a better recovery after total knee arthroplasty. J Anesth.

[5] Bonica J, Bonica J (1990). Painful disorders of the thigh and knee. The Management of Pain.

[6] Erdine S (2007). Agri, 3 Baski.

[7] Sizlan A, Atim A, Yurttas Y, Ozkan H, Bilge M, Kuyumcu M (2012). A comparison of the efficacy of bupivacaine and levobupivacaine in patient-controlled epidural analgesia for postoperative pain in patients undergoing knee arthroplasty. Joint Diseases and Related Surgery.

[8] Ashik M, Shi-Lu C, Jin YS, Hong TM, Nung LN (2010). Comparison of the different modalities of post operative analgesia in unilateral total knee arthroplasty patients. J Orthopaedics.

[9] Rasmussen S, Kramhøft MU, Sperling KP, Pedersen JH (2004). Increased flexion and reduced hospital stay with continuous intraarticular morphine and ropivacaine after primary total knee replacement: open intervention study of efficacy and safety in 154 patients. Acta Orthop Scand.

[10] Ong JCA, Lin PC, Fook-Chong SMC, Tang A, Ying YK, Keng TB (2010). Continuous infiltration of local anaesthetic following total knee arthroplasty. J Orthop Surg.

[11] Kehlet H, Andersen LO (2011). Local infiltration analgesia in joint replacement: the evidence and recommendations for clinical practice. Acta Anaesthesiol Scand.

[12] Gentili M, Houssel P, Osman M, Henel D, Juhel A, Bonnet F (1997). Intra-articular morphine and clonidine produce comparable analgesia but the combination is not more effective. Br J Anaesth.

[13] Cook TM, Tuckey JP, Nolan JP (1997). Analgesia after day-case knee arthroscopy: double-blind study of intraarticular tenoxicam, intraarticular bupivacaine and placebo. Br J Anaesth.

[14] Ivani G, Borghi B, Van Oven H (2001). Levobupivacaine. Minerva Anestesiol.

[15] Grishko V, Xu M, Wilson G, Pearsall AW (2010). Apoptosis and mitochondrial dysfunction in human chondrocytes following exposure to lidocaine, bupivacaine, and ropivacaine. J Bone Joint Surg Am.

[16] Atik OS (2012). Is single-dose local anesthetic chondrotoxic?. Joint Diseases and Related Surgery.

[17] Farkas B, Kvell K, Czompoly T, Illes T, Bardos T (2010). Increased chondrocyte death after steroid and local anesthetic combination. Clin Orthop Relat Res.

[18] Stein C, Comisel K, Haimerl E, Yassouridis A, Lehrberger K, Herz A (1991). Analgesic effect of intraarticular morphine after arthroscopic knee surgery. N Engl J Med.

[19] Kopacz DJ, Sharrock NE, Allen HW (1999). A comparison of levobupivacaine 0 125%, fentanyl 4mcg/ml, or their combination for patient-controlled epidural analgesia after major orthopedic surgery. Anesth Analg.

[20] Koltka K, Koknel-Talu G, Asik M, Ozyalcin S (2011). Comparison of efficacy of intraarticular application of magnesium, levobupivacaine and lornoxicam with placebo in arthroscopic surgery. Knee Surg Sports Traumatol Arthrosc.

